# Predictive value of transabdominal intestinal sonography in critically ill patients: a prospective observational study

**DOI:** 10.1186/s13054-019-2645-9

**Published:** 2019-11-27

**Authors:** Tao Gao, Min-Hua Cheng, Feng-Chan Xi, Yan Chen, Chun Cao, Ting Su, Wei-Qin Li, Wen-Kui Yu

**Affiliations:** 1 Research Institute of General Surgery, Jinling Hospital affiliated to Nanjing University Medical School, No.305, Zhongshan East Road, Nanjing, People’s Republic of China; 2 Department of Intensive Care Unit, Drum Tower Hospital affiliated to Nanjing University Medical School, No.321, Zhongshan Road, Nanjing, People’s Republic of China

**Keywords:** Acute gastrointestinal injury, POCUS, GUTS protocol, Abdominal hypertension, Abdominal pressure, Ultrasound

## Abstract

**Background:**

This study examined the feasibility of transabdominal intestinal ultrasonography in evaluating acute gastrointestinal injury (AGI).

**Methods:**

A total of 116 patients were included. Intestinal ultrasonography was conducted daily within 1 week after admission to the intensive care unit. Ultrasonography indicators including intestinal diameter, changes in the intestinal folds, thickness of the intestinal wall, stratification of the intestinal wall, and intestinal peristalsis (movement of the intestinal contents) were observed to determine the acute gastrointestinal injury ultrasonography (AGIUS) score. The gastrointestinal and urinary tract sonography ultrasound (GUTS) protocol score was also calculated. During the first week of the study, the gastrointestinal failure (GIF) score was determined daily. The correlations between transabdominal intestinal scores (AGIUS and GUTS) and the GIF score were analyzed to clarify the feasibility of evaluating AGI through observation of the intestine. The utility of intestinal ultrasonography indicators in predicting feeding intolerance was investigated to improve the ability of clinicians to manage AGI.

**Results:**

A total of 751 ultrasonic examinations were performed with 511 images (68%) considered to be of “good quality.” AGIUS and GUTS scores differed significantly between AGI patients (GIF score 0–2) and non-AGI patients (GIF score 3–4) (*p* < 0.001). Both scores correlated positively with GIF score (*r* = 0.54, *p* < 0.001; *r* = 0.66, *p* < 0.001). These ultrasonography indicators could predict feeding intolerance, with an area under the receiver operating characteristic curve of 0.60 (0.48–0.71; intestinal diameter), 0.76 (0.67–0.85; intestinal folds), 0.71 (0.62–0.80; wall thickness), 0.77 (0.69–0.86; wall stratification), and 0.78 (0.68–0.88; intestinal peristalsis). Compared to patients with a normal rate of peristalsis (5–10/min), patients with abnormal peristalsis rates (< 5/min or > 10/min) have increased risk for feeding intolerance (16/83 vs. 25/33, *p* < 0.001).

**Conclusions:**

The transabdominal intestinal ultrasonography represents an effective means for assessing gastrointestinal injury in critically ill patients. Intestinal ultrasonography indicators, especially the degree of intestinal peristalsis, may be used to predict feeding intolerance.

**Trial registration:**

ClinicalTrial.gov, NCT03589248. Registered 04 July 2018—retrospectively registered.

## Introduction

Gastrointestinal dysfunction is common among critically ill patients [1, 2] and leads to malnutrition, metabolic abnormalities, and internal environmental disturbances [3–5]. Thus, evaluating the degree of gastrointestinal dysfunction is of some clinical significance.

Many studies have evaluated acute gastrointestinal injury (AGI) in critically ill patients using tools such as Reintam’s gastrointestinal failure (GIF) score system [3] or the four-grade system [6], which are mainly based on gastrointestinal symptoms, feeding intolerance, and intra-abdominal hypertension (IAH). However, the occurrence of feeding intolerance or gastrointestinal symptoms depends partly on the subjective feelings of patients, and it is difficult to be predicted before the application of an intestinal protocol. IAH, which is affected by abdominal wall compliance, intra-abdominal capacity, and baseline intra-abdominal pressure (IAP) [7–9], does not always accurately represent the degree of AGI [10]. The use of biomarkers (e.g., enterohormones, citrulline, intestinal fatty acid-binding protein, d-lactate) remains controversial [11, 12].

Science 2013, point-of-care ultrasonography (POCUS) has been widely recommended to apply in the medical management algorithm for IAH and abdominal compartment syndrome (ACS), identifying and evacuating intraluminal contents or intra-abdominal lesions [13–15]. Since then, POCUS is indicated to be a modern stethoscope for all intensive care unit (ICU) patients [16, 17], as well as is described as an important diagnostic and therapeutic tool in IAH management [18, 19]. It has also been used to evaluate gastric emptying [20] or to assess duodenogastric reflux and the placement of nasogastric or nasoenteric tubes [21–23]. Whether ultrasonography could be used to predict AGI remains unclear and is a hotspot of research in the ICU.

Recently, ultrasonography was reported for daily evaluation of critically ill patients and a gastrointestinal and urinary tract sonography (GUTS) protocol was performed based on ultrasonography indicators to grade AGI [24]. However, there are several limitations of GUTS protocol as follows. First, few studies about the clinical application of GUTS protocol have been performed and its validity and reliability for the prediction of AGI still require more clinical research to confirm. Second, the GUTS protocol includes evaluation not only of the intestine but also of the stomach, IAP, and bladder. The performance of such evaluations is complicated in clinical practice. Herein, we aim to evaluate the validity of GUTS protocol for the prediction of AGI and to investigate the feasibility of using intestinal ultrasonography alone for the prediction of AGI in critically ill patient, which is a simplification of GUTS protocol. As patients may suffer feeding intolerance even after the effective management of IAH and gastric issues, an intestinal assessment is needed. Thus, we also evaluated the predict value of intestinal ultrasonography indicators in feeding intolerance. The present study aims to identify an approach with accuracy in predicting AGI and in the management of critically ill patients.

## Materials and methods

### Study subjects

This prospective, observational study was conducted in a 22-bed surgical ICU at the General Surgical Department of Jinling Hospital (affiliated with the Hospital of Nanjing University Medical School), which mainly admits patients with severe complications after severe trauma and surgery. Patients were recruited from 1 July 2016 to 1 January 2018. The protocol was approved by the Human Ethics Committee of Jinling Hospital, and informed consent was obtained from all patients or from their relatives.

Patients were screened for eligibility within 24 h of admission to the ICU. The inclusion criteria were as follows: (1) age > 18 years and (2) Acute Physiology and Chronic Health Evaluation II (APACHE II) score > 8. The exclusion criteria were as follows: (1) uncontrolled mesenteric vascular disease, (2) uncontrolled chronic organ dysfunction, (3) advanced cancers, and (4) any terminal-stage diseases.

### Standardized performance of US

**Fig. 1:**
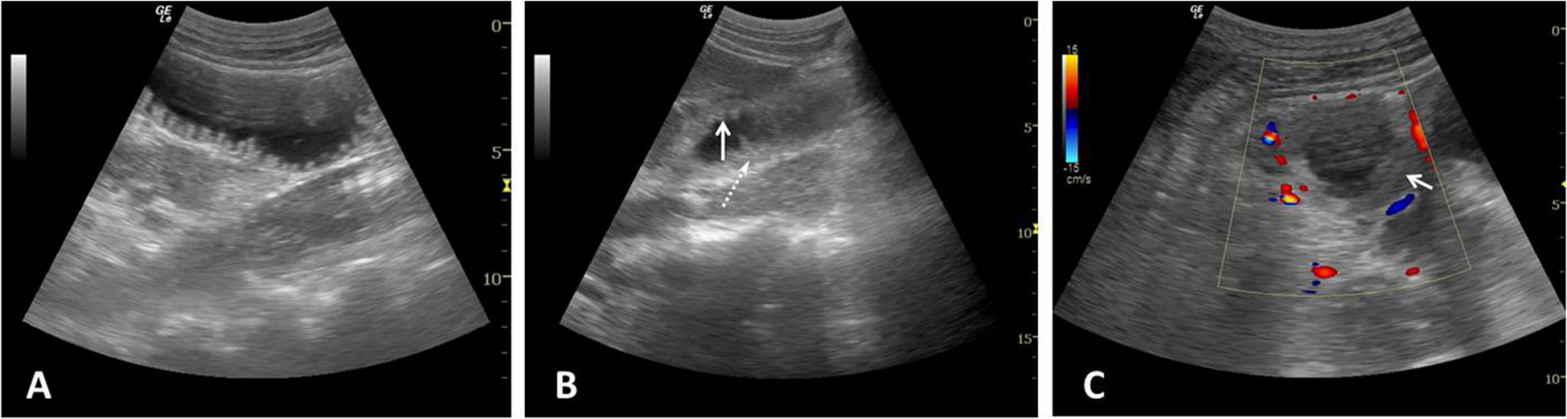
Examples of intestinal US images showing increased intestinal diameter, shortened intestinal folds, thickened intestinal walls, and stratified intestinal walls. **a** Increased intestinal diameter. **b** Shortened intestinal folds (solid arrow) and thickened intestinal walls (dotted arrow). **c** Stratified intestinal wall (solid arrow)

Two experienced attending doctors from the ICU who often perform ultrasonography (without assistance) in critically ill patients performed all ultrasonography within 1 week of a patient’s admission to the ICU. For all patients, ultrasonography was performed during the hours of 8:30–10:00 AM. Before the study, both intensivists had undergone 6 h of practical training in intestinal ultrasonography (≥ 10 cases). Patients were placed in the supine position. The abdomen was divided into four quadrants by crossing the anterior median line and umbilical horizontal line. Each region was screened with a curvilinear probe (2–5 MHz, LOGIQe, GE Healthcare, Wuxi, China). While performing the ultrasonography examination, operators classified conditions as “good quality,” “poor quality,” or “impossible.” Next, operators inspected the diameter of the intestinal canal (Fig. 1a), changes in intestinal folds (e.g., shortened, decreased, Fig. 1b), thickness of the intestinal wall (Fig. 1b), stratification of the intestinal wall (Fig. 1c), intestinal peristalsis, and movement of intestinal contents. We measured one point in each quadrant and obtained four parameters in total. The average values of four measurements (for the four quadrants of the intestine) were recorded and used to calculate the AGI ultrasonography (AGIUS) score (Table 1).

**Table 1:** Intestinal ultrasound score (AGIUS score)

	0	1	2
The diameter of the intestine	• < 3 cm, without changes in the intestine folds	• ≥ 3 cm or changes in the intestine folds	• ≥ 3 cm and changes in the intestine folds
The thickness of the intestine	• < 3 mm, without the stratified intestinal wall	• ≥ 3 mm or with stratified intestinal wall	• ≥ 3 mm and with stratified intestinal wall
Intestinal peristalsis	• 5–10/min, with transmission of intestine contents	• < 5/min or > 10/min	• No peristalsis, or without transmission of intestine contents

### Nutrition protocols

If no contraindication for enteral nutrition was present, enteral feeding was initiated 24–48 h after ICU admission. The initial infusion rate was 20 mL/h. Feeding tolerance was defined as no discomfort or abdominal distention, diarrhea, or severe reflux (vomiting, gastric residual volume ≥ 300 mL in 6 h) after enteral nutrition application, or symptoms of feeding intolerance that were relieved by treatment. Feeding intolerance was defined as the interruption of enteral nutrition because of a gastrointestinal issue (severe abdominal distention, diarrhea, vomiting, gastric residual volume ≥ 300 mL in 6 h, or subjective discomfort) [3, 25, 26]. Feeding tolerance was assessed 6 h after the initiation of enteral nutrition. If the patient reached feeding tolerance, the rate was incrementally increased by 10 mL/h until the enteral nutrition infusion rate reached the target (50 mL/h). In the case of feeding intolerance, the rate was decreased by 10 mL/h. The infusion rate was adjusted again according to the results of reassessment after 6 h. For patients at low nutritional risk (Nutritional Risk Screening 2002 [NRS-2002] < 3), we set a target caloric intake of 20 kcal/kg/day for the first week without supplemental parenteral nutrition, even in the case of feeding intolerance. For patients at high nutritional risk (NRS-2002 ≥ 3), we set a target caloric intake of 25–30 kcal/kg/day for the first week, with supplemental parenteral nutrition in the case of feeding intolerance. The target caloric intake for all patients was 25–30 kcal/kg/day over the following weeks, with supplemental parenteral nutrition if there was feeding intolerance. In the case of delayed gastric emptying, we added prokinetics to prevent pyloric bile reflux and protect the gastric mucosa (metoclopramide, 10 mg q8h, i.m.) and a postpyloric feeding route.

IAP was measured via the bladder (with patients in the supine position) using the closed-loop system repeated-measurements technique [27]. IAP was measured at least twice a day when the values recorded were normal (< 12 mmHg) and at least four times per day if the IAP was elevated above 12 mmHg. IAH and ACS were defined according to the recommendations of the World Society of the Abdominal Compartment Syndrome as well as the working group on abdominal problems of the European Society of Intensive Care Medicine [13–16].

### Data collection

Data pertaining to baseline characteristics, including age, gender, body mass index, cause of ICU admission, serum lactate, APACHE II scores, and Sequential Organ Failure Assessment (SOFA) score, were collected within the first 24 h of ICU admission. SOFA score and AGIUS score were collected or recorded daily within the first week after admission to ICU. GIF score was calculated on a daily basis during the first week of ICU admission [3] (Table 2). AGI (GIF score 0–2) patients and non-AGI (GIF score 3–4) patients were compared. AGIUS score was recorded daily during the first week after ICU admission. GUTS score was determined daily during the first week after ICU admission [24]. The GUTS score entered into the medical record was a modified version that reflected the following indices: small bowel diameter, peristalsis, IAP, and abdominal perfusion pressure (APP) (Table 3). Other data collected included intravenous fluid volume, urine output, hematocrit, gastric residual volume, mechanical ventilation (number of episodes and duration of each), PaO2/FiO_2_, platelet count, total bilirubin, mean arterial pressure (MAP), serum creatinine, use of vasoactive drugs, and number of renal replacement therapies. APP was calculated as MAP minus IAP [28]. Fluid overload within the first week was defined as cumulative fluid balance divided by baseline admission body weight (if > 10%) [29]. The primary outcomes were 28-day mortality and length of the ICU stay.

**Table 2:** Reintam’s gastrointestinal failure (GIF) score

Points	Clinical symptomatology
0	Normal gastrointestinal function
1	Enteral feeding < 50% of calculated needs or no feeding 3 days after abdominal surgery
2	Food intolerance (enteral feeding not applicable due to high gastric aspirate volume, vomiting, bowel distension, or severe diarrhea) or IAH
3	Food intolerance and IAH
4	Abdominal compartment syndrome

**Table 3:** Gastrointestinal and urinary tract sonography protocol (GUTS) score

Grade 0	Grade 1	Grade 2	Grade 3	Grade 4
• SBD < 20 mm • Peristalsis present	• SBD < 20 mm • Peristalsis absent or non-effective	• SBD 20–30 mm • Peristalsis absent, augmented, or non-effective • IAP 12–15 mmHg	• SBD > 30 mm • Peristalsis absent • IAP 16–20 mmHg • APP < 60 mmHg	• SBD > 30 mm • Peristalsis absent • IAP > 20 mmHg • APP < 60 mmHg

Our modified GUTS score was quoted from the detailed version presented in reference [24], which includes > 10 parameters

*SBD* small bowel diameter, *IAP* intra-abdominal pressure, *APP* abdominal perfusion pressure

### Statistical analyses

Patients with good ultrasonography conditions (≥ 3 times) were eligible for analysis. Maximum scores were calculated as the maximum of the individual values collected for each patient within the first week. Mean scores were calculated as the average of daily maximum values for all patients included in the study. Statistical analyses were performed using SPSS 16.0 software (SPSS, Chicago, IL). Data are expressed as means ± standard deviations, medians (interquartile range), or frequencies (percentages). Continuous variables were compared with Student’s *t* test or the Mann-Whitney *U* test when appropriate, whereas categorical variables were compared with the *χ*^2^ test or Fisher’s exact test when appropriate. The Spearman correlation test was used to analyze the correlations of ranked variables. Receiver operating characteristic (ROC) curves were used to determine the likelihood ratios for the capacity of the GIF score, AGIUS score, GUTS protocol score, SOFA score, and AGIUS score in combination with SOFA to predict 28-day mortality. Statistical significance was defined as *p* < 0.05. Univariate and multivariate logistic regression analyses were undertaken to examine the effects of variables on 28-day mortality and on AGIUS score. The results are reported as adjusted odds ratio of death with corresponding 95% confidence intervals.

## Results

### Clinical and demographic characteristics of patients

**Fig. 2:**
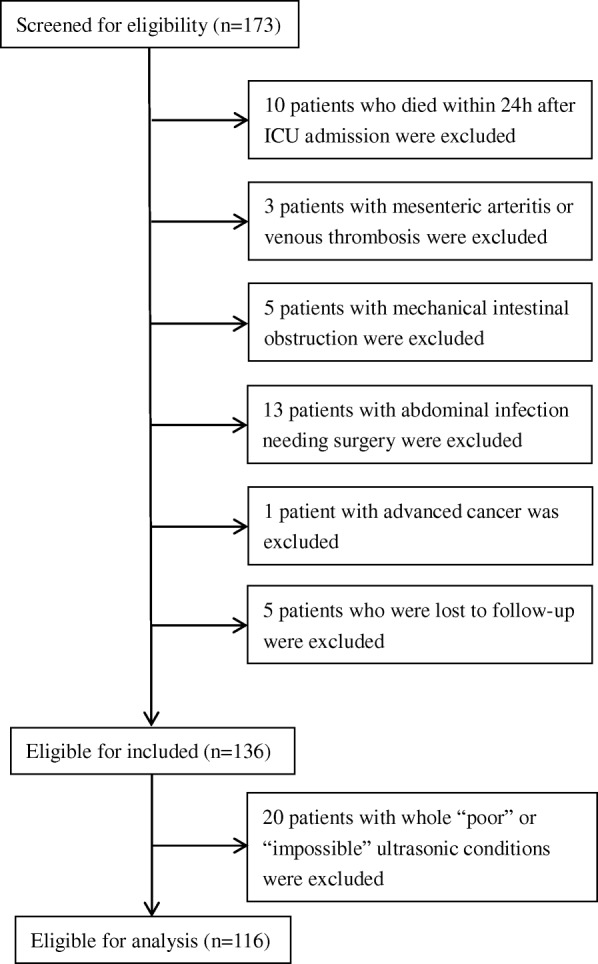
Enrollment flowchart

A total of 136 patients were considered as eligible for inclusion, and 116 were included in the final analysis (Fig. 2). Regarding the 20 ineligible patients, 14 patients were excluded because of postoperative abdominal gas, 2 patients because of unclear vision caused by the presence of drainage tubes, 2 patients because of abdominal wall defects, 1 patient because of subcutaneous abdominal wall gas, and 1 patient because of abdominal obesity. A total of 751 ultrasonic examinations were performed on all 116 patients. There were 113 (15%) examinations classified as involving “impossible” conditions. In most of these cases, gas impeded penetration of the ultrasonography. Of the remaining conditions (85%), 511 samples were classified as “good quality” (68%) and 127 as “poor quality” (17%). The corresponding clinical and demographic characteristics are shown in Table 4. Overall 28-day mortality was 36.2% (42/116). There were 46 patients in low nutritional risk group and 70 patients in the high nutritional risk group. The numbers of delivered and prescribed calories were 1105 (320–1149) and 1281 (1115–1421) kcal (*p* < 0.001) in the low nutritional risk group, and were 1685 (1458–1847) and 1778 (1496–1898) kcal (*p* = 0.19) in the high nutritional risk group. Ninety-three (80.2%) patients were successfully delivered with the total target number of calories. Sixty-one (52.6%) patients, including 38 patients in the high malnutrition risk group and 23 patients in the low malnutrition risk group, achieved the enteral nutrition target within 1 week.

**Table 4:** Characteristics of non-AGI and AGI patients

	Total (*n* = 116)	Non-AGI (*n* = 80)	AGI (*n* = 36)	*t/Z/χ*	*p* value
Age (year)	53.4 ± 20.6	53.9 ± 21.6	52.2 ± 18.5	0.41^a^	0.68
Male/female, *n* (%)	62/54	42/38	20/16	0.09^c^	0.76
BMI (kg/m^2^)	22.3 ± 3.9	22.1 ± 3.9	22.5 ± 4.0	0.49^a^	0.63
Cause of ICU admission					
Major trauma, *n* (%)	59 (50.9)	43 (53.8)	16 (44.4)	0.86^c^	0.35
Complications after surgery, *n* (%)	45 (38.8)	30 (37.5)	15 (41.7)	0.18^c^	0.67
Others, *n* (%)	12 (10.3)	7 (8.8)	5 (13.9)	0.71^c^	0.40
Serum lactate at onset (mmol/L)	2.4 ± 1.0	2.3 ± 1.0	2.5 ± 0.9	1.21^a^	0.23
APACHE II on ICU admission	15.0 [11.0–17.0]	15.0 [11.0–17.0]	14.5 [13.0–18.0]	1.83^b^	0.78
SOFA score on ICU admission	10.0 [8.0–13.0]	9.5 [8.0–13.0]	10.0 [9.3–13.0]	5.14^b^	0.48
Mechanical ventilation, *n* (%)	62 (53.5)	36 (45.0)	26 (72.2)	7.40^c^	0.007
PaO2/FiO_2_ (mmHg)	206.5 ± 21.5	209.8 ± 20.7	199.1 ± 21.6	2.54^a^	0.01
Platelet count (× 10^9^/L)	133.7 ± 19.7	137.11 ± 19.7	126.11 ± 17.7	2.87^a^	0.005
Total bilirubin (mmol/L)	70.1 ± 32.8	54.1 ± 22.7	105.8 ± 22.0	11.46^a^	< 0.001
MAP (mmHg)	75.0 ± 6.2	75.2 ± 5.6	74.3 ± 7.4	0.76^a^	0.45
Vasopressor support, *n* (%)	59 (50.9)	35 (43.8)	24 (66.7)	5.22^c^	0.02
Norepinephrine, *n* (%)	59 (50.9)	35 (43.8)	24 (66.7)	5.22^c^	0.02
Epinephrine, *n* (%)	7 (6.0)	2 (2.5)	5 (13.9)	5.68^c^	0.02
Dobutamine, *n* (%)	3 (2.6)	0 (0)	3 (8.3)	6.84^c^	0.009
Norepinephrine dose (μg/kg/min)	0.06 [0.03–0.15]	0.04 [0.03–0.06]	0.15 [0.09–0.24]	5.22^b^	< 0.001
At least two drugs, *n* (%)	9 (7.8)	2 (2.5)	7 (19.4)	9.96^c^	0.002
Serum creatinine (μmol/L)	94.0 [61.5–131.8]	80.0 [50.5–107.3]	162.0 [108.5–200.8]	18.64^b^	< 0.001
Renal replacement therapy, *n* (%)	34 (29.3)	19 (23.8)	15 (41.7)	3.85^c^	0.05
Intra-abdominal pressure (mmHg)	10.6 ± 6.3	8.4 ± 5.5	15.5 ± 5.3	6.57^a^	< 0.001
Abdominal perfusion pressure (mmHg)	64.3 ± 8.3	66.9 ± 7.8	58.8 ± 6.6	5.41^a^	< 0.001
Intra-abdominal hypertension, *n* (%)	78 (67.2)	43 (53.8)	35 (97.2)	21.30^c^	< 0.001
Feeding intolerance, *n* (%)	41 (35.3)	5 (6.3)	36 (100)	95.49^c^	< 0.001
Duration of mechanical ventilation (days)	3.0 [0.0–12.8]	1.0 [0–7.0]	12.0 [0–14.8]	4.48^b^	< 0.001
AGIUS score	2.0 [1.0–3.0]	2.0 [1.0–3.0]	4.0 [3.0–4.0]	5.85^b^	< 0.001
GUTS score	3.0 [2.0–3.0]	3.0 [2.0–3.0]	3.0 [3.0–3.0]	4.60^b^	< 0.001
Length of ICU stay (days)	9.5 [6.0–18.0]	8.0 [5.0–14.5]	17.0 [8.8–21.0]	3.38^b^	0.001
Length of hospital stay (days)	17.0 [11.0–23]	14.0 [10.0–20.8]	21.0 [19.0–25.8]	3.75^b^	< 0.001
28-day mortality, *n* (%)	42 (36.2)	14 (17.5)	28 (77.8)	39.06^c^	< 0.001
ICU mortality, *n* (%)	37 (31.9)	12 (15.0)	25 (69.4)	38.21^c^	< 0.001
Hospital mortality, *n* (%)	41 (35.3)	14 (17.5)	27 (75)	35.92^c^	< 0.001

Variables including PaO2/FiO2, platelet count, total bilirubin, norepinephrine dose, serum creatinine, and duration of mechanical ventilation were reported as the average values during stay in the ICU. Variables including mechanical ventilation, vasopressor support, and renal replacement therapy were reported as the number of patients receiving these treatments during stay in the ICU. Variables including MAP, intra-abdominal pressure, and abdominal perfusion pressure were reported as the average values of the first week after ICU admission. Variables including AGIUS score and GUTS score were reported as the highest values recorded during the first week after ICU admission. Variables including intra-abdominal hypertension and feeding intolerance were reported as the number of patients of the first week after ICU admission

*p* value represents the difference between non-AGI patients and AGI patients

Continuous data are expressed as median [Q1; Q3] or mean ± standard

*BMI* body mass index

^a^Student’s *t* test

^b^Mann-Whitney *U*test

^c^*χ*^2^ test or Fisher’s exact test

### Analysis of GIF score, SOFA score, AGIUS score, and GUTS score

**Fig. 3:**
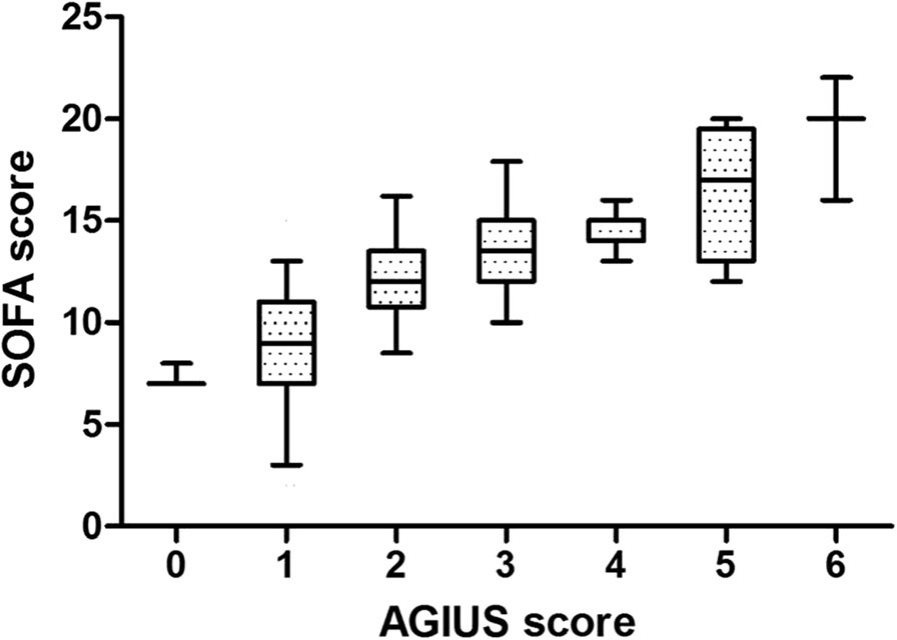
The evolution of SOFA with increasing AGIUS scores

The evolution of GIF scores within the first week is shown in Additional file 4: Figure S1. GIF scores and patient numbers are shown in Additional file 1: Table S1. On average, AGI (GIF score 3–4) occurred on day 2.5 (2.0–3.0). A positive correlation was found between GIF score and SOFA score (*r* = 0.62, *p* < 0.001) during the first week. The characteristics of AGI patients and non-AGI patients are listed in Table 4. The evolution of AGIUS scores within the first week is shown in Additional file 5: Figure S2. AGIUS scores and patient numbers are shown in Additional file 2: Table S2. AGIUS score differed significantly between AGI patients and non-AGI patients (4.0 [3.0–4.0] vs. 2.0 [1.0–3.0], *p* < 0.001). A positive correlation was found between AGIUS score and GIF score (*r* = 0.54, *p* < 0.001). Increases in AGIUS scores were associated with increases in SOFA scores (Fig. 3).

**Fig. 4:**
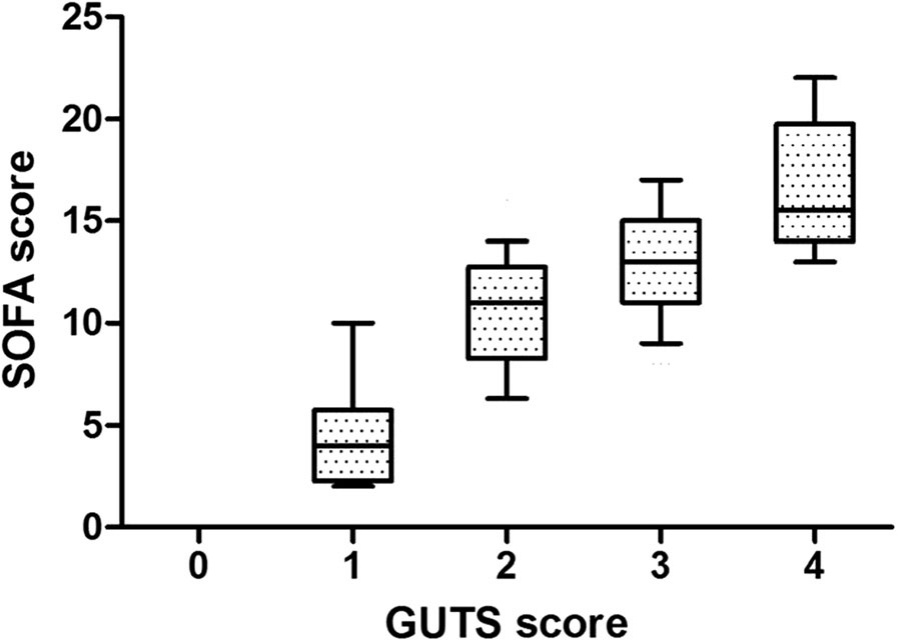
The evolution of SOFA with increasing GUTS scores

The evolution of GUTS scores within the first week is shown in Additional file 6: Figure S3. GUTS scores and patient numbers are shown in Additional file 3: Table S3. A correlation was found between GIF score and GUTS score (*r* = 0.66, *p* < 0.001). SOFA score increased with GUTS score, as shown in Fig. 4.

### Characteristics of non-IAH and IAH patients

IAH is an important factor that may result in AGI. The difference between patients with IAH and without IAH is shown in Table 5. Upon applying ROC curve analysis to identify the ultrasonography items in IAH patients, the area under the curves (AUC) was 0.51 (0.40–0.62) (intestinal diameter), 0.61 (0.51–0.72) (intestinal folds), 0.56 (0.45–0.68) (wall thickness), 0.58 (0.47–0.69) (stratified wall), and 0.39 (0.29–0.49) (intestinal peristalsis).

**Table 5:** Characteristics of non-IAH and IAH patients

	Total (*n* = 116)	Non-IAH (*n* = 38)	IAH (*n* = 78)	*t/Z/χ*^*2*^	*p* value
Cumulative fluid balance within 1 week (L)	5.0 [3.4–6.7]	4.1 [2.1–5.5]	5.5 [3.8–7.2]	0.21^b^	0.01
Fluid balance on day 1 (L)	1.6 [0.4–2.4]	1.4 [0.5–2.3]	1.7 [0.3–2.5]	0.73^b^	0.50
Fluid balance on day 2 (L)	1.1 [0.4–2.2]	0.9 [0.1–1.7]	1.5 [0.5–2.2]	0.31^b^	0.05
Fluid balance on day 3 (L)	1.0 [0.7–1.4]	0.8 [0.5–1.2]	1.1 [0.8–1.4]	0.07^b^	0.004
Fluid balance on day 4 (L)	0.6 [− 0.6–1.3]	0.5 [− 0.8–1.2]	0.6 [− 0.5–1.3]	0.06^b^	0.43
Fluid balance on day 5 (L)	0.8 [− 0.3–1.7]	0.7 [− 0.3–1.4]	0.7 [− 0.3–1.8]	2.04^b^	0.40
Fluid balance on day 6 (L)	0.1 [− 0.1–0.5]	− .05 [− 0.4–0.5]	0.2 [− 0.1–0.5]	5.26^b^	0.01
Fluid balance on day 7 (L)	0.1 [− 0.4–0.4]	0.0 [− 0.4–0.4]	0.1 [− 0.4–0.4]	0.16^b^	0.14
Urine output (mL/kg day)	14.2 [7.9–24.4]	20.3 [13.5–27.8]	10.7 [6.3–22.9]	2.80^b^	0.001
Serum creatinine (μmol/L)	94.0 [61.5–131.8]	83.0 [40.8–103.5]	108.0 [71.5–165.0]	9.73^b^	< 0.001
Fluid overload, *n* (%)	37 (31.9)	8 (21.1)	29 (37.2)	3.06^c^	0.08
Hematocrit (%)	35.4 ± 4.3	38.1 ± 3.1	34.1 ± 4.2	2.84^a^	< 0.001
Gastric residual volume (mL/day)	521.4 ± 183.3	378.3 ± 208.2	532.4 ± 179.5	4.02^a^	< 0.001
ACS, *n* (%)	5 (4.3)	0 (0)	5 (6.4)	2.52^c^	0.17
Vasopressor support, *n* (%)	59 (50.9)	18 (47.4)	41 (52.6)	0.14^c^	0.60
Norepinephrine dose (μg/kg/min)	0.06 [0.03–0.15]	0.04 [0.03–0.07]	0.13 [0.04–0.24]	3.24^b^	0.001
Mechanical ventilation, *n* (%)	62 (53.4)	17 (44.7)	45 (57.7)	0.30^c^	0.19
Duration of mechanical ventilation (days)	3.0 [0–12.8]	1.0 [0–7.2]	5.5 [0–14.0]	6.13^b^	0.03
SOFA score	12.0 [10.0–14.0]	9.5 [6.0–12.0]	13.0 [11.0–15.0]	2.39^b^	< 0.001
GIF score	2.0 [1.0–3.0]	1.0 [1.0–2.0]	2.0 [2.0–3.0]	6.21^b^	< 0.001
AGIUS score	2.0 [1.0–3.0]	1.5 [1.0–3.0]	3.0 [1.0–4.0]	2.27^b^	0.002
GUTS score	3.0 [2.0–3.0]	2.0 [2.0–2.3]	3.0 [3.0–3.0]	6.69^b^	< 0.001
Intestinal wall thickness (mm)	2.7 [2.4–2.9]	2.6 [2.6–3.0]	2.7 [2.5–2.9]	1.29^b^	0.49
Changes in the intestinal folds, *n* (%)	42 (36.2)	8 (21.1)	34 (43.6)	5.62^c^	0.02
Stratified intestinal walls, *n* (%)	58 (50)	15 (39.5)	43 (55.1)	2.51^c^	0.11
Intestinal peristalsis, frequency	6.0 [4.0–8.0]	7.0 [6.0–8.0]	6.0 [4.0–8.0]	1.87^b^	0.07
Intestinal diameter (cm)	2.6 [2.4–2.8]	2.6 [2.5–2.7]	2.6 [2.4–2.8]	0.34^b^	0.88

Variables including serum creatinine, norepinephrine dose, and duration of mechanical ventilation were reported as the average values during stay in the ICU. Variables including vasopressor support and mechanical ventilation were reported as the number of patient during stay in the ICU. Variables including urine output, hematocrit, gastric residual volume, SOFA score, intestinal wall thickness, intestinal peristalsis, and intestinal diameter were reported as the average values of the first week after ICU admission. Variables including GIF score, AGIUS score, and GUTS score were reported as the highest values recorded during the first week after ICU admission. Variables including fluid overload, ACS, changes in the intestinal folds, and stratified intestinal walls were reported as the number of patients during the first week after ICU admission

*p* value represents difference between non-IAH patients and IAH patients

Continuous data are expressed as median [Q1; Q3] or mean ± standard

^a^Student’s *t* test

^b^Mann-Whitney *U* test

^c^*χ*^2^ test or Fisher’s exact test

### Comparison of ultrasonography indicators in patients with vs. without feeding intolerance

Feeding intolerance is another sign of AGI. When patients with feeding intolerance (41/116, 35.3%) were compared to patients without feeding intolerance (75/116, 64.7%), there were significant differences in terms of intestinal wall thickness (2.8 [2.7–3.1] vs. 2.6 [2.4–2.8] mm, *p* = 0.001), changes in the intestinal folds (28/41 vs. 13/75, *p* < 0.001), number of stratified intestinal walls (35/41 vs. 23/75, *p* < 0.001), and risk for disturbances of intestinal peristalsis (4.0 [3.0–7.0] vs. 7.0 [6.0–9.0], *p* < 0.001), while no significant difference was found for intestinal diameter (2.7 [2.5–2.8] vs. 2.6 [2.4–2.7] cm, *p* = 0.06). Use of ROC curve analysis to identify the ultrasonography items in feeding intolerance yielded AUC values of 0.60 (0.48–0.71) (intestinal diameter), 0.76 (0.67–0.85) (intestinal folds), 0.71 (0.62–0.80) (wall thickness), 0.77 (0.69–0.86) (stratified wall), and 0.78 (0.68–0.88) (intestinal peristalsis), respectively. In total, 24 out of 29 (82.8%) patients with intestinal peristalsis frequency < 5/min experienced feeding intolerance within the first week, compared with 16 out of 83 (19.3%) patients with intestinal peristalsis frequency of 5–10/min, and 1 of 4 (25.0%) patients with intestinal peristalsis frequency > 10/min. In addition, when comparing patients with a normal rate of peristalsis (5–10/min) to those with abnormal peristalsis rates (< 5/min or > 10/min), the probability of feeding intolerance was significantly lower in the former group (16/83 vs. 25/33, *p* < 0.001). Patients with abnormal peristalsis were more likely to have received treatment with prokinetics than patients with normal peristalsis (30/33 vs. 32/83, *p* < 0.001).

### Stratified analysis for patients with low vs. high AGIUS scores

In the ROC curve analysis of predicting 28-day mortality, a cutoff value of 2.0 for AGIUS score had a sensitivity of 85.7% and a specificity of 71.6%. Therefore, we used an AGIUS score of 2 to stratify the clinical data. There were 59 patients with low AGIUS scores (≤ 2) and 57 patients with high AGIUS scores (> 2). A significant difference in 28-day mortality was found between groups (6/59 vs. 36/57, *p* < 0.001). In addition, patients with low AGIUS scores had lower rate of mechanical ventilation, lower renal replacement therapy rate, lower serum lactate level, lower SOFA scores, shorter duration of mechanical ventilation, and shorter duration of ICU stay (Table 6).

**Table 6:** Stratified analysis for patients with low vs. high AGIUS scores

	Total (*n* = 116)	AGIUS score ≤ 2 (*n* = 59)	AGIUS > 2 (*n* = 57)	*t/Z/χ*^2^	*p* value
Cumulative fluid balance within 1 week (L)	5.0 [3.4–6.7]	4.7 [2.6–5.8]	5.6 [4.0–7.3]	0.13^b^	0.008
Serum lactate at onset (mmol/L)	2.4 ± 1.0	2.5 ± 0.9	2.3 ± 1.0	0.13^a^	0.36
Vasopressor support, *n* (%)	59 (50.9)	25 (42.4)	34 (59.7)	3.46^c^	0.06
Norepinephrine dose (μg/kg/min)	0.06 [0.03–0.15]	0.03 [0.03, 0.04]	0.12 [0.07–0.22]	4.92^b^	< 0.001
Mechanical ventilation, *n* (%)	62 (53.4)	26 (44.1)	36 (63.2)	4.25^c^	0.04
Duration of mechanical ventilation (days)	3.0 [0–12.8]	1.0 [0–7.0]	9.0 [0–14.0]	12.18^b^	0.001
Serum creatinine (μmol/L)	94.0 [61.5–131.8]	70.0 [41.0–91.0]	128.0 [94.0–185.5]	13.25^b^	< 0.001
Renal replacement therapy, *n* (%)	34 (29.3)	9 (15.3)	25 (43.9)	11.45^c^	0.001
Feeding intolerance, *n* (%)	41 (35.3)	6 (10.2)	35 (61.4)	33.30^c^	< 0.001
Intra-abdominal hypertension, *n* (%)	78 (67.2)	35 (59.3)	43 (75.4)	3.42^c^	0.06
GIF score	2.0 [1.0–3.0]	2.0 [1.0–2.0]	3.0 [2.0–3.0]	4.18^b^	< 0.001
SOFA score	12.0 [10.0–14.0]	10.0 [8.0–12.0]	14.0 [12.5–15.5]	2.44^b^	< 0.001
Length of ICU stay (days)	9.5 [6.0–18.0]	8.0 [5.0–15.0]	14.0 [6.5–20.0]	2.45^b^	0.01
28-day mortality, *n* (%)	42 (36.2)	7 (11.9)	35 (61.4)	30.80^c^	< 0.001
ICU mortality, *n* (%)	37 (31.9)	5 (8.5)	32 (56.1)	30.32^c^	< 0.001
Hospital mortality, *n* (%)	41 (35.3)	7 (11.9)	34 (59.7)	28.97^c^	< 0.001

Variables including vasopressor support, mechanical ventilation, and renal replacement therapy were reported as the number of patients during stay in the ICU. Variables including serum creatinine, norepinephrine dose, and duration of mechanical ventilation were reported as the average values recorded during stay in the ICU. Variables including intra-abdominal hypertension and feeding intolerance were reported as the number of patients during the first week after ICU admission. SOFA score was reported as the average of the first week after ICU admission. GIF score were reported as the highest values observed during the first week after ICU admission

*p* value represents difference between low AGIUS patients and high AGIUS patients

Continuous data are expressed as median [Q1; Q3] or mean ± standard

^a^Student’s *t* test

^b^Mann-Whitney *U* test

^c^*χ*^2^ test or Fisher’s exact test

### ROC curve analysis for use of AGIUS, GIF, or GUTS score in predicting 28-day mortality

**Fig. 5:**
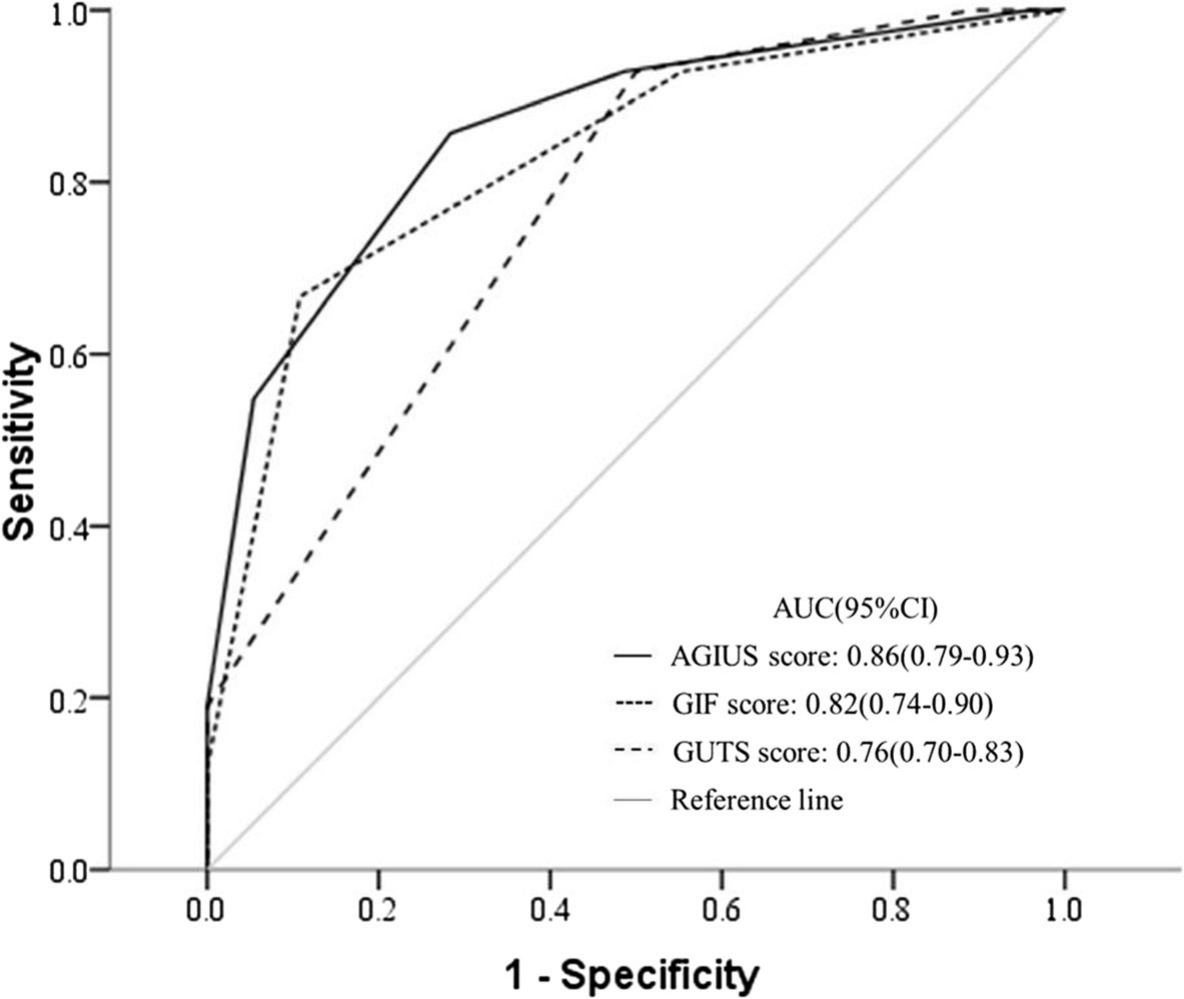
ROC curve analysis for use of AGIUS, GIF, or GUTS score to predict 28-day mortality. Variables included in analyses were the maximum value within the first week

ROC curve analysis was used to identify the sensitivities and specificities of the AGIUS score, GUTS score, and GIF score in predicting 28-day mortality, as shown in Fig. 5. The AUC of ROC analysis revealed that the AGIUS score had higher predictive value than the GIF score for predicting 28-day mortality (0.86 (0.79–0.93) vs. 0.82 (0.74–0.90), *p* = 0.10). The AUC of the ROC was larger for AGIUS score than for GUTS score (0.86 (0.79–0.93) vs. 0.76 (0.70–0.83), *p* = 0.03). Combining the AGIUS score with the SOFA score yielded higher AUC for predicting 28-day mortality than did use of SOFA score alone (0.89 (0.83–0.95) vs. 0.86 (0.80–0.93), *p* = 0.12).

### Univariate and multivariate analyses of AGIUS score and 28-day mortality

Univariate logistic regression showed that intravenous fluid volume, serum lactate level, norepinephrine dose, duration of mechanical ventilation, serum creatinine, feeding intolerance, IAP, GIF score, and SOFA score were significantly associated with AGIUS score. Multivariate logistic regression analysis identified mechanical ventilation, renal replacement therapy, GIF score, and SOFA score as independent predictors of high AGIUS score (Table 7).

**Table 7:** Univariate and multivariate analyses of 28-day mortality in patients

	Univariate		Multivariate	
	OR (95% CI)	*p* value	OR (95% CI)	*p* value
Age (year)	1.00 (0.98–1.02)	0.68		
BMI (kg/m^2^)	1.09 (0.98–1.20)	0.11		
Serum lactate at onset (mmol/L)	1.22 (1.10–1.34)	< 0.001	1.38 (1.15–1.61)	< 0.001
Vasopressor support, *n* (%)	4.57 (2.37–6.77)	< 0.001	1.67 (1.17–2.16)	0.003
Mechanical ventilation, *n* (%)	3.69 (2.38–5.00)	< 0.001		
Renal replacement therapy, *n* (%)	2.53 (1.46–4.61)	< 0.001		
Feeding intolerance, *n* (%)	2.32 (1.27–3.65)	< 0.001		
Intra-abdominal pressure (mmHg)	1.22 (1.12–1.33)	< 0.001		
SOFA score	1.42 (1.33–1.58)	< 0.001	1.53 (1.07–2.79)	0.02
AGIUS score	2.55 (1.36–3.89)	< 0.001	1.71 (1.48–2.73)	0.001

Variables included in regression were age, BMI, serum lactate at onset, vasopressor support (yes/no during stay in the ICU), mechanical ventilation (yes/no during stay in the ICU), renal replacement therapy (yes/no during stay in the ICU), feeding intolerance (yes/no during the first week), intra-abdominal pressure (highest value recorded during the first week), SOFA score (highest value recorded during the first week), and AGIUS score (highest value recorded during the first week)

*BMI* body mass index, *OR* odds ratio, *CI* confidence intervals

Univariate and multivariate analyses of 28-day mortality are shown in Table 8. Multivariate logistic regression analysis identified serum lactate, use of vasopressor support, SOFA score, and AGIUS score as independent predictors of 28-day mortality.

**Table 8:** Univariate and multivariate analyses of AGIUS scores

	Univariate		Multivariate	
	OR (95% CI)	*p* value	OR (95% CI)	*p* value
Cumulative fluid balance within 1 week (L)	1.22 (1.05–1.42)	0.01		
Serum lactate at onset (mmol/L)	0.83 (0.56–1.28)	0.35		
Vasopressor support, *n* (%)	2.01 (0.96–4.21)	0.06		
Mechanical ventilation, *n* (%)	2.18 (1.03–3.32)	0.04	2.70 (1.38–4.02)	0.01
Duration of mechanical ventilation (days)	1.18 (1.06–1.32)	0.004		
Renal replacement therapy, *n* (%)	1.34 (1.20–1.48)	0.001	1.67 (1.34–2.01)	< 0.001
Feeding intolerance, *n* (%)	2.05 (1.18–2.94)	< 0.001		
Intra-abdominal pressure (mmHg)	1.09 (1.02–1.16)	0.007		
GIF score	2.26 (1.17–3.46)	< 0.001	2.70 (1.79–4.86)	0.002
SOFA score	1.51 (1.29–1.78)	< 0.001	1.76 (1.51–2.23)	0.003

Variables included in regression were cumulative fluid balance within 1 week, serum lactate at onset, vasopressor support (yes/no within the first week), mechanical ventilation (yes/no within the first week), duration of mechanical ventilation (days within the first week), renal replacement therapy (yes/no within the first week), feeding intolerance (yes/no during the first week), intra-abdominal pressure (highest value recorded during the first week), GIF score (highest value recorded during the first week), and SOFA score (highest value recorded during the first week)

*OR* odds ratio, *CI* confidence intervals

## Discussion

In our study, we found that transabdominal intestinal ultrasonography may be used to evaluate AGI and that intestinal ultrasonography indicators may be used to predict feeding intolerance in critically ill patients. Numerous efforts have been made to assess AGI, including some based on the use of ultrasonography, especially in patients with delayed gastric emptying [30–34]. Here, we explored the application of transabdominal intestinal ultrasonography in the assessment of AGI. The results demonstrated the feasibility of this approach and indicated that intestinal ultrasonography indicators may be used to design an individualized approach to feeding management in critically ill patients.

### Predictive value of intestinal sonography for AGI

We found transabdominal intestinal ultrasonography scores (AGIUS, GUTS) were correlated with AGI grade and GIF score, confirming the possibility of intestinal ultrasonography in predicting AGI. Intestinal ultrasonography examinations provide objective and quantifiable indicators such as intestinal thickness, intestinal diameter, and degree of intestinal peristalsis. We found that the indicators initially assessed in the clinic were feeding intolerance, IAP, and symptoms; this approach remains controversial. Because of a lack of universally accepted definitions, the strength of the relationship between feeding intolerance and mortality varies substantially between studies [35]. The occurrence of feeding intolerance depends strongly on feeding practices (route, formula, and rate) and on the subjective feelings of patients, which can vary. The monitoring of IAP is recommended as a routine treatment for critically ill patients with AGI [36]. However, gastrointestinal dysfunction can lead to IAH and vice versa. IAP is not a direct indicator of gastrointestinal function, which may confuse the relationship between gastrointestinal symptoms and IAP [37]. In that sense, intestinal ultrasonography has certain advantages over other methods used previously by practitioners.

### Predictive value of intestinal sonography for feeding intolerance

The incidence of feeding intolerance was 35.5% in our study, compared with 30.5% in another study [26]. Our study demonstrated the value of intestinal ultrasonography indicators in predicting feeding intolerance. As known, feeding intolerance is commonly encountered during feeding critically ill patients. Although the early administration of normocaloric enteral nutrition has been associated with favorable clinical outcomes [38, 39], the presence of feeding intolerance increases the risk for aspiration pneumonia or enterogenic infection [40–43]. A recent systematic review showed that hypocaloric enteral nutrition, compared with full-energy nutrition, did not significantly affect morbidity or mortality [44]. While if there is no feeding intolerance, continuous trophic feeding can result in the delayed recovery of total enteral nutrition. Thus, appropriate initiation and temporal adjustments to the protocol are essential. However, we cannot predict tolerance of the initial or adjusted protocol. Therefore, the identification of feeding intolerance before the initiation of enteral nutrition would make sense. Because good correlations between feeding intolerance and intestinal ultrasonography indicators were found, we speculate that ultrasonography may be used to predict feeding intolerance before nutrition is provided. Thus, in patients with normal peristalsis, enteral nutrition could be initiated without delay, allowing for early adjustment of the feeding protocol, while in patients with abnormal peristalsis, more time may be required before it is safe to initiate enteral nutrition and to adjust the feeding protocol. By predicting feeding intolerance, we may decrease the risk of enteral nutrition and facilitate the implementation of an individualized nutrition protocol. This possibility warrants further study.

### Predictive value of intestinal sonography for IAH/ACS

As reported previously, there were significant differences between IAH and non-IAH patients in cumulative fluid balance within 1 week, urine output, hematocrit, gastric residual volume, norepinephrine dose, and mechanical ventilation [45]. Unfortunately, no intestinal ultrasonography indicator showed significant predictive value for IAH/ACS, although the combined intestinal ultrasonography score had predictive value. The predictive value of intestinal sonography for IAH/ACS requires further study.

### Selection of indicators for intestinal ultrasound

Transabdominal ultrasonography is a reliable detection method used in patients with Crohn’s disease, ileus, celiac disease, intussusception, infectious enteritis, tumors, or ischemic/hemorrhagic conditions of the small bowel [46, 47]. Nevertheless, few studies have applied it for AGI in critically ill patients, especially intestinal ultrasonography. Thus, the optimal approach to the selection of intestinal indicators and the use of those indicators for AGI assessment in critically ill patients remains unclear.

**Fig. 6:**
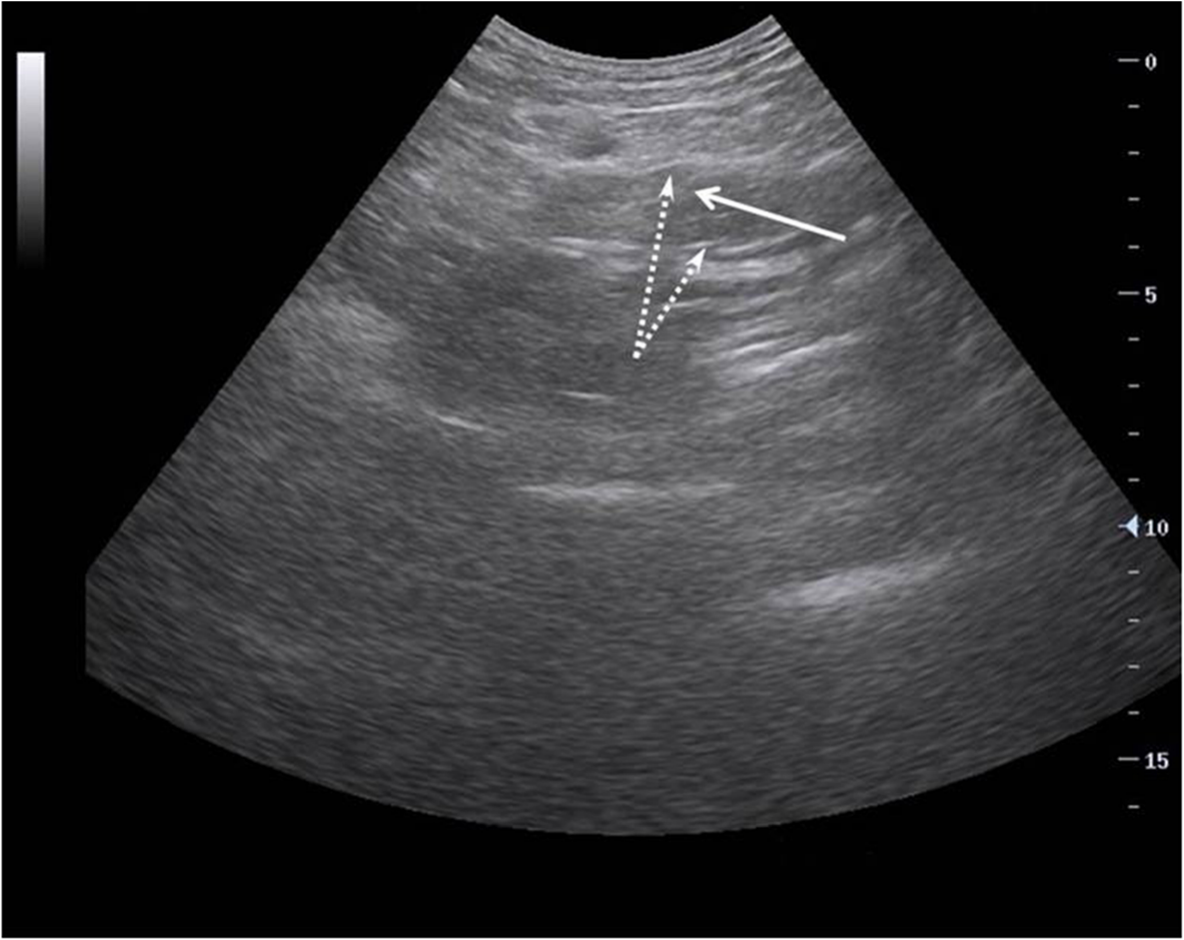
Normal transabdominal intestinal ultrasonography. This is a 36-year-old male. The intestine was screened with a curvilinear probe (5 MHz). The image shows normal intestinal diameter (< 2 cm), normal intestinal thickness (< 2 mm, dotted arrow), and normal folds (long without edema, solid arrow)

Five indicators were selected for use in our study: intestinal thickness, stratification of the intestinal wall, intestinal peristalsis, changes to intestinal folds, and intestinal diameter. By using transabdominal ultrasound, clinicians can detect thickened colon walls, diagnose inflammatory colitis [48], and measure the thickness of the intestinal wall in patients with *Yersinia* enteritis [49]. Fluid overload can result in bowel and intestinal edema, as well as second- and third-space free fluid [29, 50, 51]. Capillary leak syndrome leads to the accumulation of interstitial fluid, which can present as increased intestinal thickness or even obvious stratifications. We therefore speculate that the thickness and stratification of the intestinal wall could be used to evaluate the degree of intestinal injury. Intestinal peristalsis is a basic physiological function that is crucial for the maintenance of normal digestion and absorption. Prokinetics can ameliorate feeding intolerance in critically ill patients [52]. Early-stage enteritis is associated with enhanced motility [53]. Regardless of whether motility is increased or decreased, abnormal peristalsis indicates gastrointestinal dysfunction, so we used intestinal peristalsis as a parameter. The normality of gastrointestinal function also depends on fold integrity. Ultrasound is used to detect changes in the intestinal folds in patients with celiac disease [54, 55]. The present study showed that abnormal intestinal folds were more common in critically ill patients (Fig. 1), compared with healthy adult (Fig. 6). Intestinal dilation is multifactorial and mainly depends on the capacity of the intestine to deal with its contents (absorption or discharge). In serious AGI cases, the worsened gastrointestinal capacity to deal with intestinal contents is expressed as abnormal intestinal diameter. We therefore included intestinal diameter as an indicator in this study. In our study, we analyzed the prognostic value of these indicators in predicting outcomes among patients with IAH and feeding intolerance in order to determine the feasibility of applying these indicators in clinical practice.

Rather than using a single indicator, we applied two scoring systems: GUTS and AGIUS, both of which incorporate multiple indicators due to the complexities of intestinal function. The GUTS score includes intestinal morphological and functional indicators, as well as local hemodynamic parameters and organ perfusion indicators. The AGIUS score includes intestinal morphological and functional indicators. Although both scoring systems have demonstrated predictive value in the treatment of AGI, there were some differences. We found that the correlation between GUTS score and GIF score was stronger than that between AGIUS score and GIF score. This may be because the weight of IAP was substantial for the GUTS score since only four parameters were included, and IAP is one of the important parameters in the GIF score. The inaccuracy of the GUTS score should also be noted since the GUTS score in our study included only four parameters while the original GUTS protocol includes more than ten parameters [24]. The limited parameters may impair the prognostic strength of the GUTS score. Although there are differences in the indicators used to yield two scores, the AGIUS score is part of the GUTS score and is used specifically for the evaluation of small intestine-related indicators. That is to say, the two scores may be supplementary to each other when predicting AGI. Further study will be necessary to identify the optimal intestinal ultrasonography indicators (and corresponding ranges) for use in the evaluation of patients with AGI.

### Study limitations

There were limitations to our study. First, transabdominal ultrasonography is influenced by examination conditions, such as intra-abdominal gas or abdominal wall defects. Twenty patients were excluded from analysis because of these effects. Even among patients included in the analysis, the proportion of “good” pictures was only 68% of the total. These results indicate that we cannot complete the ultrasonography evaluation at one time, and ultrasonography cannot completely replace other methods for the evaluation of gastrointestinal function. Second, a major cause of enhanced intestinal wall thickness is mesenteric vascular lesions. In critically ill patients, coagulopathy and microthrombosis are common pathophysiological changes that may affect mesenteric vessels [56]. In our study, although patients with obvious mesenteric vascular lesions were excluded, and the average intestinal thickness of four regions was calculated, we were not sure whether intestinal microvascular lesions caused this thickening. In future studies, the use of vaso-enhanced ultrasonography may minimize this heterogeneity. Third, sonography is a highly device- and operator-dependent method. Regarding device dependency, convex and linear probes use different frequencies. The linear probe is more accurate than the convex probe in the discrimination of organization. Because we did not include mesenteric vessel parameters in our study, we applied a convex probe only to acquire a large detection area and depth. Regarding operator dependency, although we constructed a 7-grade score depending on intestinal ultrasonography, there were still some subjective parameters, such as “changes in intestinal folds” and “stratified intestinal wall,” which require the correct interpretation of experienced operators. Objective clear cutoffs, as in the GUTS protocol, are essential.

Additionally, our nutritional protocol differed slightly from the guidelines. One issue is the incorporation of early enteral nutrition guidelines in our enteral nutrition protocol [57]. Considering the probable effects of different enteral nutrition infusion rates on intestinal diameter and feeding tolerance, we set a maximum infusion rate (50 mL/h) in order to minimize the variation in feeding rate caused by different weights. Another issue is the use of supplemental parenteral nutrition in critically ill patients. According to the American Society for Parenteral and Enteral Nutrition guidelines, for patients at high nutritional risk, exclusive parenteral nutrition should be performed as soon as possible following ICU admission when enteral nutrition is not feasible [38]. Several recent studies have considered the rationality of supplemental parenteral nutrition [58–60]. In our study, if there was only feeding intolerance, patients at high nutrition risk received supplemental parenteral nutrition. The earliest time that supplemental parenteral nutrition was started was 48–72 h after ICU admission.

## Conclusions

Transabdominal intestinal ultrasonography represents an effective means for assessing AGI in the management of critically ill patients. In contrast to other assessment methods, transabdominal ultrasonography allows for the direct observation of intestinal morphology and function, resulting in a more targeted assessment of AGI. Intestinal ultrasonography indicators (especially intestinal peristalsis) may be used to predict feeding intolerance and to manage clinical feeding practice. Additional studies should be performed to determine the optimal intestinal ultrasonography indicators for the management of feeding practice in the treatment of critically ill patients.

## Supplementary information

**Additional file 1: Table S1.** GIF scores and patient numbers.

**Additional file 2: Table S2.** AGIUS scores and patient numbers.

**Additional file 3: Table S3.** GUTS scores and patient numbers.

**Additional file 4: Figure S1.** GIF score evolution.

**Additional file 5: Figure S2.** AGIUS score evolution.

**Additional file 6: Figure S3.** GUTS score evolution.

## Data Availability

All data generated or analyzed during this study are included in this published article. The detailed datasets used and/or analyzed during the current study are available from the corresponding author upon reasonable request.

## Abbreviations

ACS: Abdominal compartment syndrome
AGI: Acute gastrointestinal injury
AGIUS score: Acute gastrointestinal injury ultrasonography score
APACHE: Acute Physiology and Chronic Health Evaluation II
APP: Abdominal perfusion pressure
AUC: Area under the curve
GIF: Gastrointestinal failure
GUTS protocol: The gastrointestinal ultrasound protocol
IAH: Intra-abdominal hypertension
IAP: Intra-abdominal pressure
ICU: Intensive care unit
MAP: Mean arterial pressure
NRS-2002: Nutritional Risk Screening 2002
POCUS: Point-of-care ultrasound
ROC: Receiver operating characteristic
SOFA: Sequential Organ Failure Assessment Scores
