# Supplementary material for: Predictive value of transabdominal intestinal sonography in critically ill patients: a prospective observational study
Source: Crit Care. 2019 Nov 27;23:378. doi: 10.1186/s13054-019-2645-9 (PMC6880579; doi:10.1186/s13054-019-2645-9)
Supplement: Supplementary file 1 — Additional file 1: Table S1. GIF scores and patient numbers. [file 13054_2019_2645_MOESM1_ESM.docx]

Supplementary Table 1 GIF scores and patient numbers

|  |  | Day 1 | Day 2 | Day 3 | Day 4 | Day 5 | Day 6 | Day 7 | Max score |
| --- | --- | --- | --- | --- | --- | --- | --- | --- | --- |
| n |  | 116 | 116 | 115 | 114 | 112 | 111 | 111 | 116 |
| GIF score | 0 | 16 (13.8) | 20 (17.2) | 15 (13.0) | 17 (14.9) | 12 (10.7) | 15 (13.5) | 15 (13.5) | 0 (0.0) |
|  | 1 | 61 (52.6) | 31 (26.7) | 25 (21.7) | 24 (21.1) | 36 (32.1) | 30 (27.0) | 43 (38.7) | 36 (31.0) |
|  | 2 | 30 (25.9) | 47 (40.5) | 46 (40.0) | 47 (41.2) | 37 (33.0) | 43 (38.7) | 29 (26.1) | 44 (37.9) |
|  | 3 | 9 (7.8) | 17 (14.7) | 29 (25.2) | 25 (21.9) | 23 (20.5) | 20 (18.0) | 20 (18.0) | 31 (26.7) |
|  | 4 | 0 (0.0) | 1 (0.9) | 0 (0.0) | 1 (0.9) | 4 (3.6) | 3 (2.7) | 4 (3.6) | 5 (4.3) |

Data in table are expressed as frequencies (percentages).

Maximum scores were calculated as the maximum of the individual values collected for each patient within one week.
