# Supplementary material for: Predictive value of transabdominal intestinal sonography in critically ill patients: a prospective observational study
Source: Crit Care. 2019 Nov 27;23:378. doi: 10.1186/s13054-019-2645-9 (PMC6880579; doi:10.1186/s13054-019-2645-9)
Supplement: Supplementary file 2 — Additional file 2: Table S2. AGIUS scores and patient numbers. [file 13054_2019_2645_MOESM2_ESM.docx]

Supplementary Table 2 AGIUS scores and patient numbers

|  |  | Day 1 | Day 2 | Day 3 | Day 4 | Day 5 | Day 6 | Day 7 | Max score |
| --- | --- | --- | --- | --- | --- | --- | --- | --- | --- |
| n |  | 116 | 116 | 115 | 114 | 112 | 112 | 111 | 116 |
| AGIUS Score | 0 | 38 (32.8) | 37 (31.9) | 27 (23.5) | 14 (12.3) | 39 (34.8) | 36 (32.1) | 37 (33.3) | 3 (2.6) |
|  | 1 | 38 (32.8) | 33 (28.5) | 28 (24.3) | 33 (29.0) | 21 (18.8) | 33 (29.5) | 34 (30.6) | 38 (32.8) |
|  | 2 | 23 (19.8) | 23 (19.8) | 23 (20.0) | 22 (19.3) | 17 (15.2) | 22 (19.6) | 32 (28.8) | 18 (15.5) |
|  | 3 | 11 (9.5) | 17 (14.7) | 21 (18.3) | 28 (24.6) | 21 (18.8) | 15 (13.4) | 5 (4.5) | 30 (25.9) |
|  | 4 | 6 (5.2) | 3 (2.6) | 8 (7.0) | 12 (10.5) | 11 (9.8) | 2 (1.8) | 0 (0.0) | 19 (16.4) |
|  | 5 | 0 (0.0) | 3 (2.6) | 5 (4.3) | 3 (2.6) | 2 (1.8) | 3 (2.7) | 2 (1.8) | 5 (4.3) |
|  | 6 | 0 (0.0) | 0 (0.0) | 3 (2.6) | 2 (1.8) | 1 (0.9) | 1 (0.9) | 1 (0.9) | 3 (2.6) |

Data in table are expressed as frequencies (percentages).

The max scores were calculated as the max of the individual values for every patient within one week.
