# Supplementary material for: Predictive value of transabdominal intestinal sonography in critically ill patients: a prospective observational study
Source: Crit Care. 2019 Nov 27;23:378. doi: 10.1186/s13054-019-2645-9 (PMC6880579; doi:10.1186/s13054-019-2645-9)
Supplement: Supplementary file 3 — Additional file 3: Table S3. GUTS scores and patient numbers. [file 13054_2019_2645_MOESM3_ESM.docx]

Supplementary Table3 GUTS scores and patient numbers

|  |  | Day1 | Day2 | Day3 | Day4 | Day5 | Day6 | Day7 | Max score |
| --- | --- | --- | --- | --- | --- | --- | --- | --- | --- |
| n |  | 116 | 116 | 115 | 114 | 112 | 112 | 111 | 116 |
| GUTS score | 0 | 2 (1.7) | 1 (0.9) | 0 (0) | 0 (0) | 1 (0.9) | 2 (1.8) | 2 (1.8) | 0 (0) |
|  | 1 | 39 (33.6) | 51(44.0) | 30(26.1) | 33 (28.9) | 52 (46.4) | 49 (43.8) | 59 (53.2) | 8 (6.9) |
|  | 2 | 52 (44.8) | 36 (31.0) | 35(30.4) | 22 (19.3) | 18 (16.1) | 22 (19.6) | 17 (15.3) | 33 (28.4) |
|  | 3 | 22 (19.0) | 23 (19.8) | 42(36.5) | 51 (44.7) | 34 (30.4) | 32 (28.6) | 27 (24.3) | 67 (57.8) |
|  | 4 | 1 (0.9) | 5 (4.3) | 8 (7.0) | 8 (7.0) | 7 (6.3) | 8 (7.1) | 6 (5.4) | 8 (6.9) |

Data in table are expressed as frequencies (percentages).

The max scores were calculated as the max of the individual values for every patient within one week
