# Supplementary figures and images for: Predictive value of transabdominal intestinal sonography in critically ill patients: a prospective observational study
Source: Crit Care. 2019 Nov 27;23:378. doi: 10.1186/s13054-019-2645-9 (PMC6880579; doi:10.1186/s13054-019-2645-9)

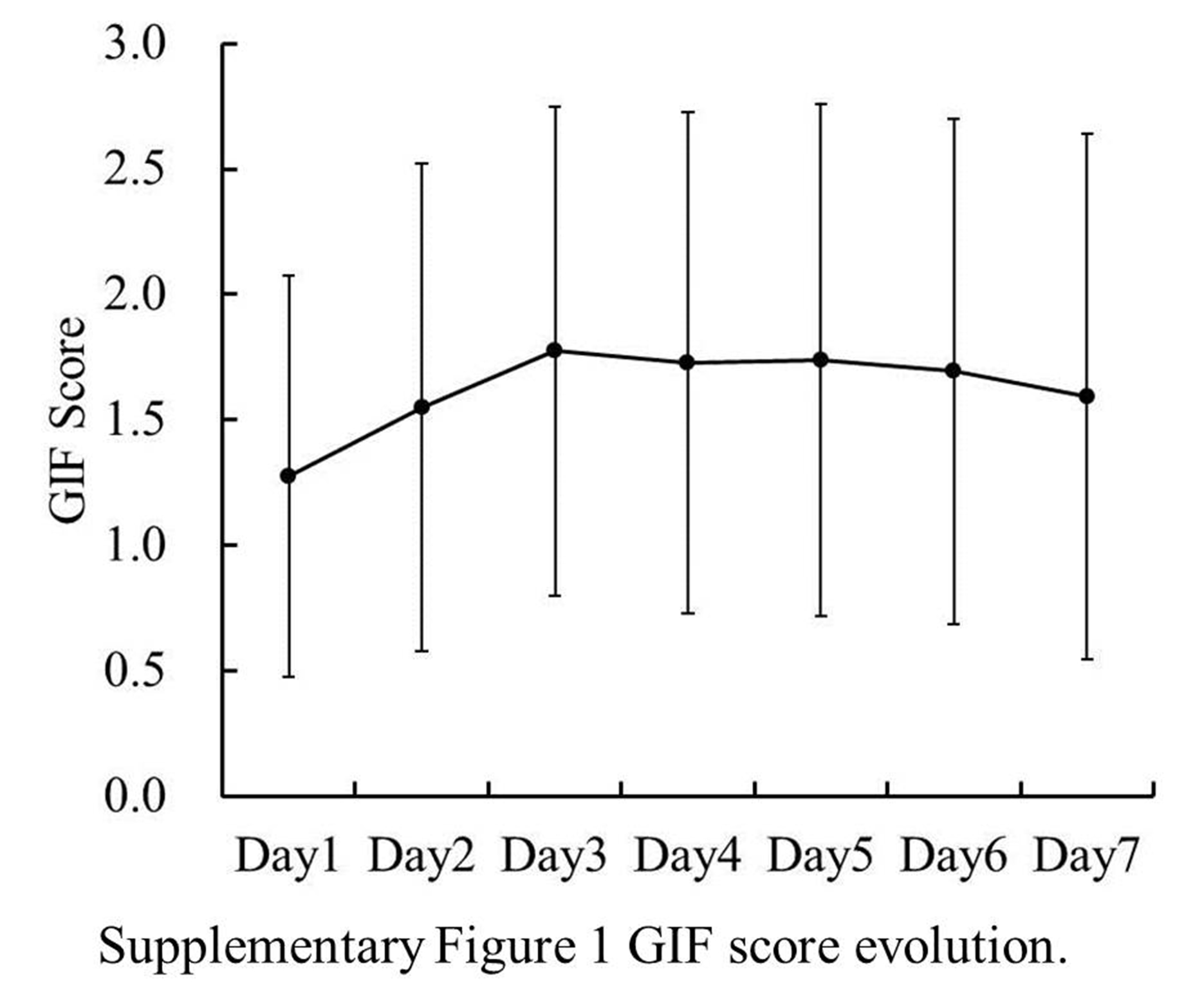

Supplement: Supplementary file 4 — Additional file 4: Figure S1. GIF score evolution. [file 13054_2019_2645_MOESM4_ESM.jpg]

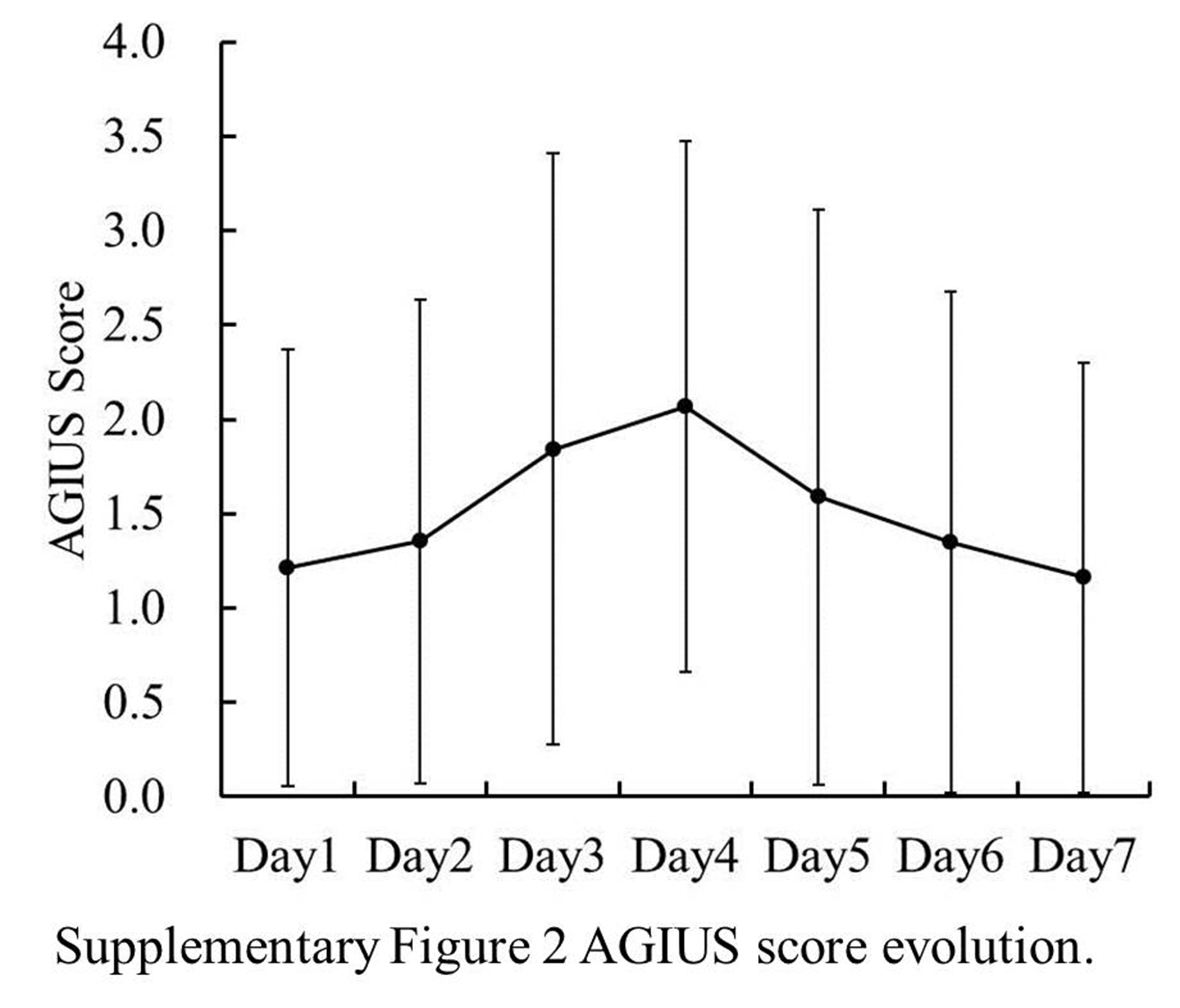

Supplement: Supplementary file 5 — Additional file 5: Figure S2. AGIUS score evolution. [file 13054_2019_2645_MOESM5_ESM.jpg]

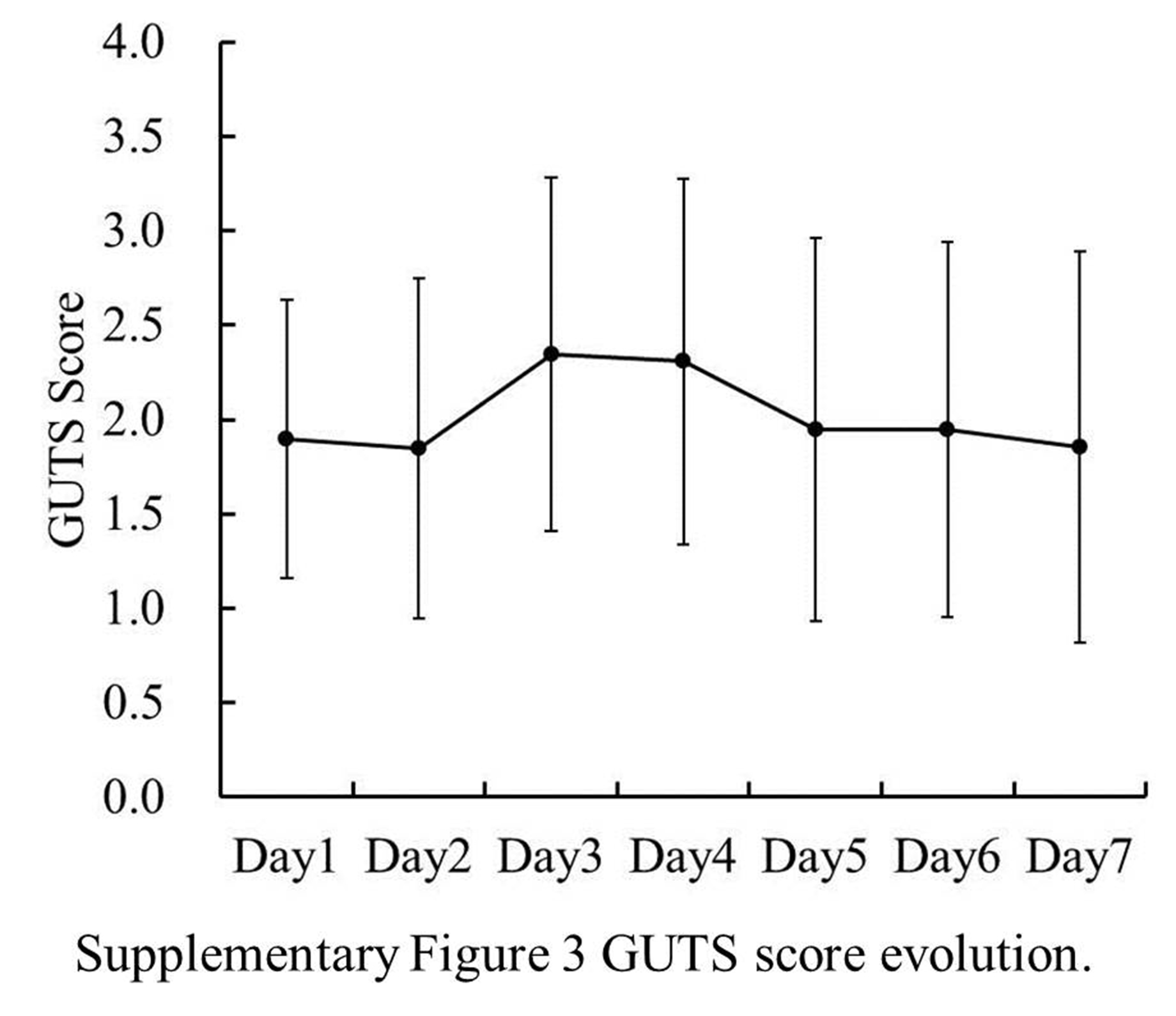

Supplement: Supplementary file 6 — Additional file 6: Figure S3. GUTS score evolution. [file 13054_2019_2645_MOESM6_ESM.jpg]
